# A 3-step approach to predict advanced fibrosis in nonalcoholic fatty liver disease: impact on diagnosis, patient burden, and medical costs

**DOI:** 10.1038/s41598-022-22767-z

**Published:** 2022-10-28

**Authors:** Takashi Kobayashi, Yuji Ogawa, Satoru Shinoda, Michihiro Iwaki, Asako Nogami, Yasushi Honda, Takaomi Kessoku, Kento Imajo, Masato Yoneda, Satoru Saito, Kouji Yamamoto, Satoshi Oeda, Hirokazu Takahashi, Yoshio Sumida, Atsushi Nakajima

**Affiliations:** 1 Department of Gastroenterology and Hepatology, Yokohama City University Graduate School of Medicine, Yokohama, Japan; 2 Gastroenterology Division, National Hospital Organization Yokohama Medical Center, Yokohama, Japan; 3 Department of Biostatistics, Yokohama City University School of Medicine, Yokohama, Japan; 4 Department of Gastroenterology, Shin-Yurigaoka General Hospital, Kawasaki, Japan; 5 Liver Center, Saga University Hospital, Saga, Japan; 6 Division of Hepatology and Pancreatology, Department of Internal Medicine, Aichi Medical University, Aichi, Japan

**Keywords:** Hepatology, Liver diseases

## Abstract

A 2-step approach, Fibrosis-4 index (FIB-4) followed by vibration-controlled transient elastography (VCTE), has been proposed to predict advanced fibrosis in patients with nonalcoholic fatty liver disease (NAFLD). We aimed to develop a novel 3-step approach for predicting advanced fibrosis. We enrolled 284 biopsy-confirmed NAFLD patients from two tertiary care centers and developed subgroups (n = 190), including 3.7% of patients with advanced fibrosis, assuming a primary care setting. In the 3-step approach, patients with intermediate-to-high FIB-4 in the first step underwent an enhanced liver fibrosis test or measurement of type IV collagen 7S domain as the second step, and VCTE was performed if the second step value was higher than the cutoff. In 284 cases, a tertiary care cohort with 36.3% advanced fibrosis, the 3-step approach showed significantly higher specificity and positive predictive value than the 2-step approach. In the subgroup with 3.7% advanced fibrosis, the 3-step approach significantly reduced the referral rate to specialists, the number of high-risk patients (i.e., liver biopsy candidates), and healthcare costs by 12.5% to 15.8%. The 3-step approach may improve the diagnostic performance to predict advanced fibrosis in NAFLD, which could lower rates of referrals to specialists, liver biopsies, and medical costs.

## Introduction

Nonalcoholic fatty liver disease (NAFLD) is becoming an increasingly common cause of liver disease, making it a major public health concern^1,2^. Hepatic fibrosis is the strongest influencing factor of NAFLD prognosis. Patients with advanced fibrosis are at high risk for cirrhosis, hepatocellular carcinoma, and cardiovascular events^3^; thus, they require referral to a specialist and additional investigations. However, unnecessary referrals will also increase specialist workload and patient medical costs owing to travel and expenses. In addition, not all patients have access to specialized facilities. Therefore, only the most appropriate patients should be recommended for referrals. Liver biopsy has also become the gold standard for diagnosing advanced fibrosis; however, it is costly, invasive, and can lead to life-threatening complications. Therefore, reducing the number of unnecessary liver biopsies is also important.

Accordingly, noninvasive tests to predict NAFLD with advanced fibrosis have developed rapidly in recent years. The fibrosis-4 index (FIB-4) is a scoring system for predicting liver fibrosis and can be calculated with a simple formula using age, aspartate aminotransferase (AST) and alanine aminotransferase (ALT) levels, and platelet count. FIB-4 divides patients into three groups: low, intermediate, and high score groups. FIB-4 is recommended as a screening test to rule out NAFLD with advanced fibrosis because it can be calculated using common laboratory test results, is low cost, has a simple formula, and has a high negative predictive value (NPV) in the low score group^4^.

In recent years, a 2-step approach combining FIB-4 and vibration-controlled transient elastography (VCTE) has been proposed to increase the positive predictive value (PPV)^5–7^. In this 2-step approach, first, FIB-4 is assessed in patients with NAFLD; patients with low FIB-4 generally do not require additional testing and can be managed in primary care. Meanwhile, patients with intermediate and high FIB-4 should undergo VCTE. If the VCTE value is higher than the cutoff, that is, the patient is considered at high risk, further tests such as liver biopsy are required. In general, this 2-step approach is designed to screen for high-risk NAFLD cases. However, although this has the benefit of being straightforward with few steps, it also has the disadvantage of requiring a reasonable number of referrals to a facility that can perform VCTE.

Among noninvasive tests that can be performed by a blood test, the enhanced liver fibrosis (ELF) test and the type IV collagen 7S domain (T4C7S) have been reported to have high diagnostic accuracy for fibrosis in NAFLD patients. The ELF score is calculated using an equation combining the concentrations of three serum markers of hepatic extracellular matrix metabolism—hyaluronic acid, tissue inhibitor of metalloproteinase-1, and N-terminal peptide of procollagen III^8^—and shows a high diagnostic ability for fibrosis in NAFLD patients, with an area under the receiver operating characteristic curve of 0.80–0.90 for advanced fibrosis^9–11^. The T4C7S is a fragment of type IV collagen and is used as a biomarker of liver fibrosis in NAFLD^12,13^. It can be measured as a single parameter in serum using a chemiluminescent enzyme immunoassay^14^. T4C7S has an area under the receiver operating characteristic curve of 0.83–0.91^15,16^.

This study aimed to develop an economical but accurate diagnostic approach that could achieve lower specialist referral rates and better follow-up for NAFLD patients in primary care clinics. Toward this goal, we focused on tests accessible in primary care clinics. The performance of a novel 3-step approach, in which patients with intermediate-to-high FIB-4 (i.e., FIB-4 higher than the lower cutoff) undergo ELF or T4C7S as a second step followed by VCTE as a third step, was compared with a conventional 2-step approach to test whether the 3-step approach reduces the number of patients referred to or undergoing liver biopsy. We also compared the medical costs of these approaches. Finally, we validated the diagnostic performance of this 3-step approach in predicting advanced fibrosis in NAFLD by referring to the liver biopsy results.

## Results

### Patient characteristics

A total of 284 patients were enrolled, of which 156 (54.9%) were women, with a mean (standard deviation; ± SD) age and body mass index (BMI) of 57.1 (± 14.0) years and 28.8 (± 4.6) kg/m^2^, respectively. Liver biopsies showed that 103 (36.3%) of the 284 patients had NAFLD with advanced fibrosis (fibrosis stage ≥ 3). The 181 patients diagnosed with fibrosis stage ≤ 2 were 54.6 (± 14.3) years old and had a BMI of 28.3 (± 4.3) kg/m^2^. The 103 patients diagnosed with fibrosis stage ≥ 3 were 61.5 (± 12.3) years old and had a BMI of 29.5 (± 5.1) kg/m^2^. The patient characteristics are shown in Table 1.

**Table 1:** Patient characteristics.

	Total patients (n = 284)	Patients with stage 0 – 2 fibrosis (n = 181)	Patients with stage 3 – 4 fibrosis (n = 103)
Age, years	57.1 (14.0)	54.6 (14.3)	61.5 (12.3)
Men	128	87	41
Women	156	94	62
BMI, kg/m^2^	28.8 (4.6)	28.3 (4.3)	29.5 (5.1)
Diabetes, n (%)	181 (63.7)	105 (58.0)	76 (73.8)
Hypertension, n (%)	166 (58.5)	97 (53.6)	69 (67.0)
Dyslipidemia, n (%)	204 (71.8)	134 (74.0)	70 (68.0)
Fast blood sugar, mg/dL	117.0 (25.9)	116.6 (28.1)	117.7 (21.8)
HbA1c, %	6.4 (1.0)	6.3 (1.1)	6.4 (0.9)
F-IRI, µU/ml	18.2 (10.2)	16.8 (9.1)	20.9 (11.7)
HOMA-IR	5.2 (3.2)	4.8 (3.0)	6.0 (3.6)
TC, mg/dL	190.0 (38.4)	196.3 (39.9)	179.3 (33.4)
HDL-C, mg/dL	49.6 (13.0)	49.9 (12.0)	49.1 (14.6)
LDL-C, mg/dL	116.3 (33.1)	121.7 (33.7)	107.1 (30.0)
TG, mg/dL	159.5 (95.7)	165.6 (104.0)	148.8 (78.1)
ALT, U/L	67.9 (44.7)	70.3 (47.9)	63.7 (38.4)
AST, IU/L	53.2 (28.9)	49.6 (28.6)	59.7 (28.3)
GGT, IU/L	80.2 (99.2)	75.0 (108.9)	89.3 (78.7)
Albumin, g/dL	4.3 (0.4)	4.3 (0.3)	4.2 (0.4)
Total bilirubin, mg/dL	0.6 (0.4)	0.8 (0.3)	0.9 (0.4)
PLT, × 10^3^/µL	196 (61)	212 (58)	167 (56)
FIB-4	2.35 (1.70)	1.82 (1.28)	3.27 (1.94)
AAR	0.90 (0.37)	0.81 (0.37)	1.05 (0.33)
APRI	1.02 (0.70)	0.83 (0.50)	1.35 (0.86)
ELF score	10.1 (1.1)	9.7 (0.9)	11.0 (0.9)
T4C7S, ng/ml	6.0 (2.0)	5.1 (1.4)	7.5 (1.9)
**VCTE**			
CAP, dB/m	290.2 (52.4)	292.8 (49.2)	285.7 (57.4)
LSM, kPa	11.3 (7.3)	8.6 (4.3)	16.1 (9.0)
Steatosis score, 1/2/3, n	136/103/45	81/68/32	55/35/13
Inflammation score, 0/1/2/3, n	11/179/79/15	8/131/38/4	3/48/41/11
Ballooning score, 0/1/2, n	118/117/48	95/67/19	23/50/29
**Fibrosis stage, n (%)**			
F0	17 (6.0)		
F1	100 (35.2)		
F2	64 (22.5)		
F3	86 (30.3)		
F4	17 (6.0)		

Data are mean (SD) unless otherwise stated. AAR, AST/ALT ratio; ALT, alanine transaminase; APRI, AST to platelet ratio; AST, aspartate aminotransferase; BMI, body mass index; CAP, controlled attenuation parameter; ELF, enhanced liver fibrosis test; F-IRL, Fasting insulin resistance index; FIB-4, Fibrosis-4 index; GGT, γ-glutamyltranspeptidase; HDL, high-density lipoproteins; HOMA-IR, Homeostatic Model Assessment of Insulin Resistance; LDL, low-density lipoprotein; LSM, liver stiffness measurement; PLT, platelet count; T4C7S, type IV collagen 7S domain; TC, total cholesterol; TG, triglycerides; VCTE, vibration-controlled transient elastography.

### Diagnostic performance of each noninvasive test

The diagnostic performances of various noninvasive tests in 284 patients, in a tertiary care cohort with 36.3% advanced fibrosis, are summarized in Supplementary Table S1 and Supplementary Fig. S1. The area under the receiver operating characteristic curve of FIB-4 was 0.768; ELF, 0.840; T4C7S, 0.863; and VCTE, 0.818. Only the last three had area under the receiver operating characteristic curves > 0.800. The cutoff for discriminating between the FIB-4 low- and intermediate-to-high-risk groups was set at 1.3, and the cutoff values for ELF and VCTE were set at 9.5 and 7.8 kPa, respectively, based on a previous study^17^. The cutoff value for T4C7S was set to 5.0 based on prior reports^18^. For ELF, the results for low cutoff and high cutoff recommended by Siemens and the results for cutoff recommended by NICE guidelines are also described^19^ (Supplementary Table S1).

### Performance of each approach in the tertiary care cohort with a high prevalence of advanced fibrosis

**Figure 1:**
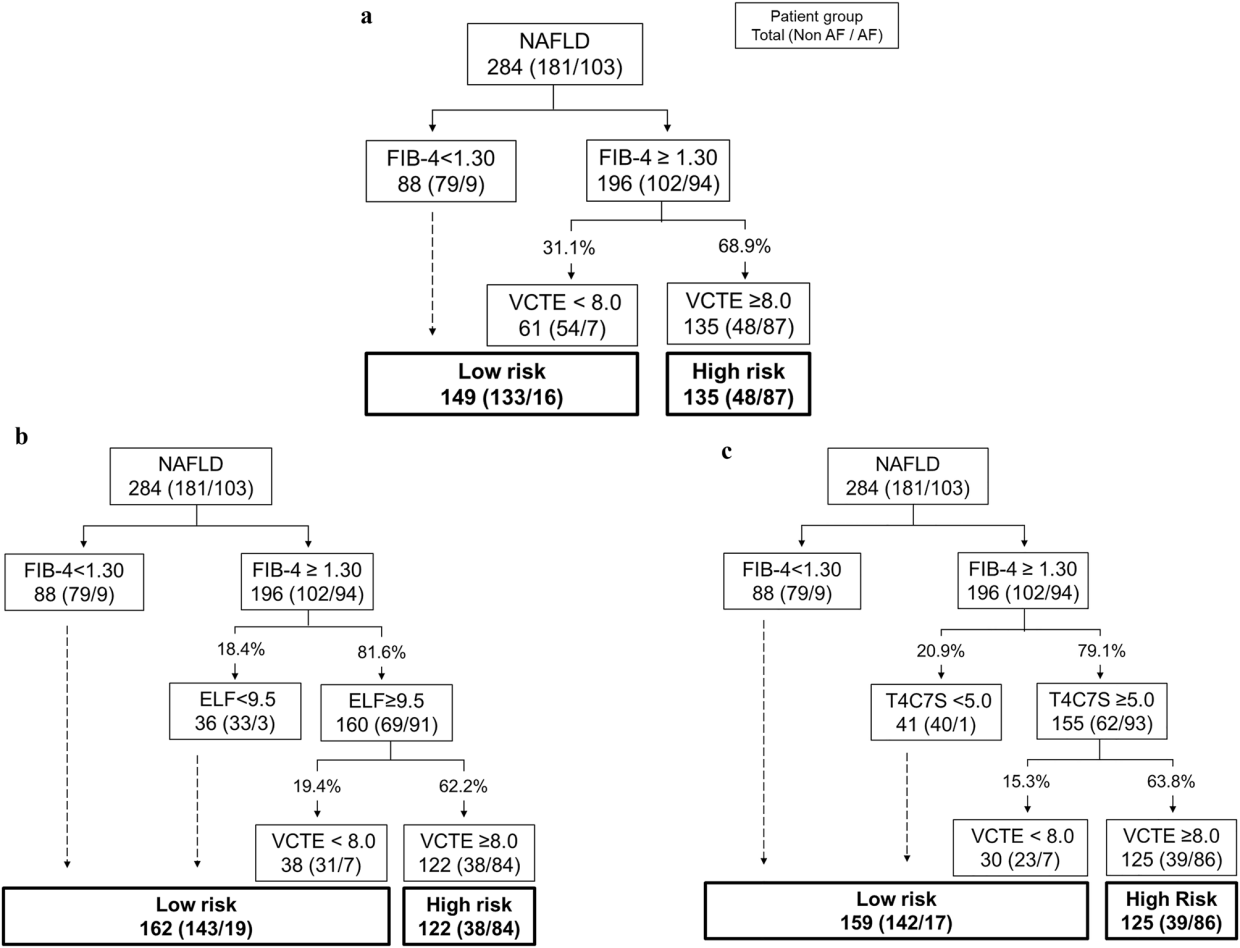
Performance of each approach in the tertiary care center cohort. Number and percentages of patients evaluated using the (**a**) 2-step approach, (**b**) 3-step approach with ELF, and (**c**) 3-step approach with T4C7S. The numbers in the boxes and parentheses are the sum of the total patients and the number of patients without advanced fibrosis/number of patients with advanced fibrosis, respectively. The numbers on the lines are the percentages among patients with a FIB-4 score of ≥ 1.30. AF, advanced fibrosis; ELF, enhanced liver fibrosis test; FIB-4, Fibrosis-4 index; T4C7S, type IV collagen 7S domain; VCTE, vibration-controlled transient elastography.

The results of the 2-step approach are shown in Fig. 1a and Table 2. The 2-step approach had a sensitivity of 84.5%; specificity, 73.5%; PPV, 64.4%; and NPV, 89.3%.

**Table 2:** Diagnostic performance of each approach for predicting advanced fibrosis in a tertiary care cohort with a high prevalence of advanced fibrosis.

	Se, %	Sp, %	PPV, %	NPV, %	Diagnosed as high risk/all cases, %
FIB-4	91.3	43.7	48.0	89.8	69.0
2-step: FIB-4–VCTE	84.5	73.5	64.4	89.3	47.5
3-step: FIB-4– ELF–VCTE	81.6	79.0	68.9	88.3	43.0
P value*	0.25	**0.002**	**0.004**	0.291	**0.000**
3-step: FIB-4– T4C7S–VCTE	83.5	78.5	68.8	89.3	44.0
P value*	1.0	**0.004**	**0.004**	1.000	**0.002**

Bold indicates significance.

ELF, enhanced liver fibrosis test; FIB-4, Fibrosis-4 index; NPV, negative predictive value; PPV, positive predictive value; Se, sensitivity; Sp, specificity; T4C7S, type IV collagen 7S domain; VCTE, vibration-controlled transient elastography.

*P-value for comparison to 2-step (FIB-4–VCTE).

The performance of the 3-step approach is shown in Fig. 1b, c and Table 2. The 3-step approach with ELF had a sensitivity of 81.6%; specificity, 79.0%; PPV, 68.9%; and NPV, 88.3%. Compared with the 2-step approach, sensitivity and NPV were not significantly different, while the specificity (P = 0.002) and PPV (P = 0.004) were significantly higher. Meanwhile, the 3-step approach with T4C7S had a sensitivity of 83.5%; specificity, 78.5%; PPV, 68.8%; and NPV, 89.3%. Again, compared with the 2-step approach, sensitivity and NPV showed no significant difference, but the specificity (P = 0.004) and PPV (P = 0.004) were significantly higher.

In addition, significantly fewer cases were considered at high risk (i.e., candidates for liver biopsy) in the 3-step approach than in the 2-step approach (47.5% for the 2-step vs. 43.0% for the 3-step ELF [P < 0.001] and 44.0% for the 3-step T4C7S [P = 0.002]).

### Performance of each approach in cohorts with 3.7% prevalence of advanced fibrosis

To simulate the patient referral rate from primary care to specialty facilities, we created subpopulations with a 3.7% prevalence of advanced fibrosis, assuming a primary care clinic setting. We further corrected bias using the bootstrap method and evaluated each approach. The results are shown in Table 3. Assuming that blood tests such as ELF and T4C7S can be performed in primary care clinics and VCTE requires referral to a specialist clinic, 57.6 ± 3.6% (95% confidence interval [CI] 50.5–64.7) of patients required referral to a specialist clinic with the 2-step approach, whereas only 40.0 ± 3.5% (95% CI 33.2–46.8) and 36.3 ± 3.4% (95% CI 29.5–43.2) of patients required referrals when using the 3-step approach with ELF and T4C7S, respectively. Both referral rates in the 3-step approach were significantly lower than those in the 2-step approach since 95% CIs of the differences remained negative. In addition, significantly fewer patients were considered high risk, that is, requiring liver biopsy, in the 3-step than in the 2-step approach [28.2 ± 3.2% in the 2-step compared with 23.2 ± 2.9% in the 3-step approach with ELF and 23.8 ± 3.0% in the 3-step approach with T4C7S (Table 3)]. Regarding diagnostic accuracy, the 3-step approach showed significantly higher specificity and a trend toward higher PPV than the 2-step one, maintaining a sensitivity of > 80% and an NPV of approximately 99% (Table 3).

**Table 3:** Diagnostic performance of each approach for predicting advanced fibrosis in cohort with a 3.7% prevalence of advanced fibrosis.

	Se, %	Sp, %	PPV, %	NPV, %	Diagnosed as high risk/ all cases, %	Referral cases/ all cases, %
FIB-4	91.2 ± 10.7 (71.4 to 100.0)	43.7 ± 3.7 (36.6 to 50.8)	5.8 ± 0.7 (4.2 to 7.1)	99.2 ± 0.9 (97.2 to 100.0)	–	-
2-step: FIB-4–VCTE	84.4 ± 13.7 (57.1 to 100.0)	73.5 ± 3.3 (67.2 to 79.8)	11.0 ± 2.0 (6.9 to 14.9)	99.2 ± 0.7 (97.7 to 100.0)	28.7 ± 3.2 (22.6 to 34.7)	57.6 ± 3.6 (50.5 to 64.7)
3-step: FIB-4–ELF–VCTE	81.5 ± 14.7 (57.1 to 100.0)	**79.0 ± 3.0** (73.2 to 84.7)	13.1 ± 2.7 (7.8 to 18.4)	99.1 ± 0.7 (97.7 to 100.0)	**23.2 ± 2.9** (17.4 to 28.9)	**40.0 ± 3.5** (33.2 to 46.8)
Difference (vs. 2-step)	− 2.9 ± 6.3 (− 14.3 to 0.0)	5.5 ± 1.7 (2.7 to 8.8)	2.1 ± 1.3 (− 0.6 to 4.7)	− 0.1 ± 0.3 (− 0.7 to 0.2)	− 5.4 ± 1.6 (− 8.9 to − 2.6)	− 17.7 ± 2.8 (− 23.2 to − 12.6)
3-step: FIB-4–T4C7S–VCTE	83.4 ± 14.0 (57.1 to 100.0)	**78.4 ± 3.1** (72.1 to 84.2)	13.1 ± 2.6 (8.0 to 18.2)	99.2 ± 0.7 (97.8 to 100.0)	**23.8 ± 3.0** (17.9 to 30.0)	**36.3 ± 3.4** (29.5 to 43.2)
Difference (vs. 2-step)	− 1.0 ± 3.7 (− 14.3 to 0.0)	5.0 ± 1.6 (2.2 to 8.2)	2.1 ± 1.0 (− 0.1 to 4.3)	0.0 ± 0.2 (− 0.7 to 0.2)	− 4.8 ± 1.5 (− 7.9 to − 2.1)	− 21.3 ± 3.0 (− 27.3 to − 15.8)

Upper line indicates mean ± SD, and lower line indicates 95% CI.

Bold letters indicate a significant difference compared to the 2-step approach.

CI, confidence interval; ELF, enhanced liver fibrosis test; FIB-4, Fibrosis-4 index; NPV, negative predictive value; PPV, positive predictive value; Se, sensitivity; Sp, specificity; T4C7S, type IV collagen 7S domain; VCTE, vibration-controlled transient elastography.

### Medical costs

The mean medical cost per person per session for each approach was calculated in the cohort with a prevalence of advanced fibrosis of 3.7%, assuming a primary care setting. In British pounds, the average cost of the 2-step approach was ￡468.6 ± 36.3 (95% CI 398.4–540.8), while that of the 3-step approach with ELF was ￡394.6 ± 35.8 (95% CI 326.2–468.2), with a cost saving of approximately 15.8% (Table 4a). The cost difference for these approaches was − 74.0 ± 19.9 (95% CI − 115.1 to − 37.6), a significant decrease since the 95% CI remained in the negative range. Furthermore, in Japanese yen, the 2-step approach cost ￥1216.8 ± 68.7 (95% CI 1083.5–1353.0), while the 3-step approach with T4C7S cost ￥1065.2 ± 71.3 (95% CI 927.9–1207.9), reducing the cost by approximately 12.5% (Table 4b). The cost difference for these approaches was − 151.6 ± 37.8 (95% CI − 228.8 to − 81.2), and since the 95% CI was in the negative range, this was also a significant decrease.

**Table 4:** Mean total cost per person at 3.7% prevalence of advanced fibrosis.

	Mean	95% CI
**(a) 2-step approach and the 3-step ELF (in British pound)**		
2-step: FIB-4–VCTE	468.6 (36.3)	398.4 to 540.8
3-step: FIB-4–ELF–VCTE	394.6 (35.8)	326.2 to 468.2
Difference	− 74.0 (19.9)	− 115.1 to − 37.6
**(b) 2-step approach and the 3-step-T4C7S (in Japanese yen)**		
2-step: FIB-4–VCTE	1216.8 (68.7)	1083.5 to 1353.0
3-step: FIB-4–T4C7S–VCTE	1065.2 (71.3)	927.9 to 1207.9
Difference	− 151.6 (37.8)	− 228.8 to − 81.2

Data are means (SD) except for 95% CI.

Units are in (a) British pound and (b) Japanese yen.

CI, confidence interval; ELF, enhanced liver fibrosis test; FIB-4, Fibrosis-4 index; T4C7S, type IV collagen 7S domain; VCTE, vibration-controlled transient elastography.

## Discussion

For the prediction of advanced fibrosis in NAFLD patients, compared with the widely accepted 2-step approach, the novel 3-step approach significantly increased the specificity and PPV without significantly decreasing the sensitivity or NPV when used in a cohort with a high prevalence of advanced fibrosis. In addition, the 3-step approach significantly reduced the number of cases diagnosed as high risk, that is, those requiring liver biopsy.

**Figure 2:**
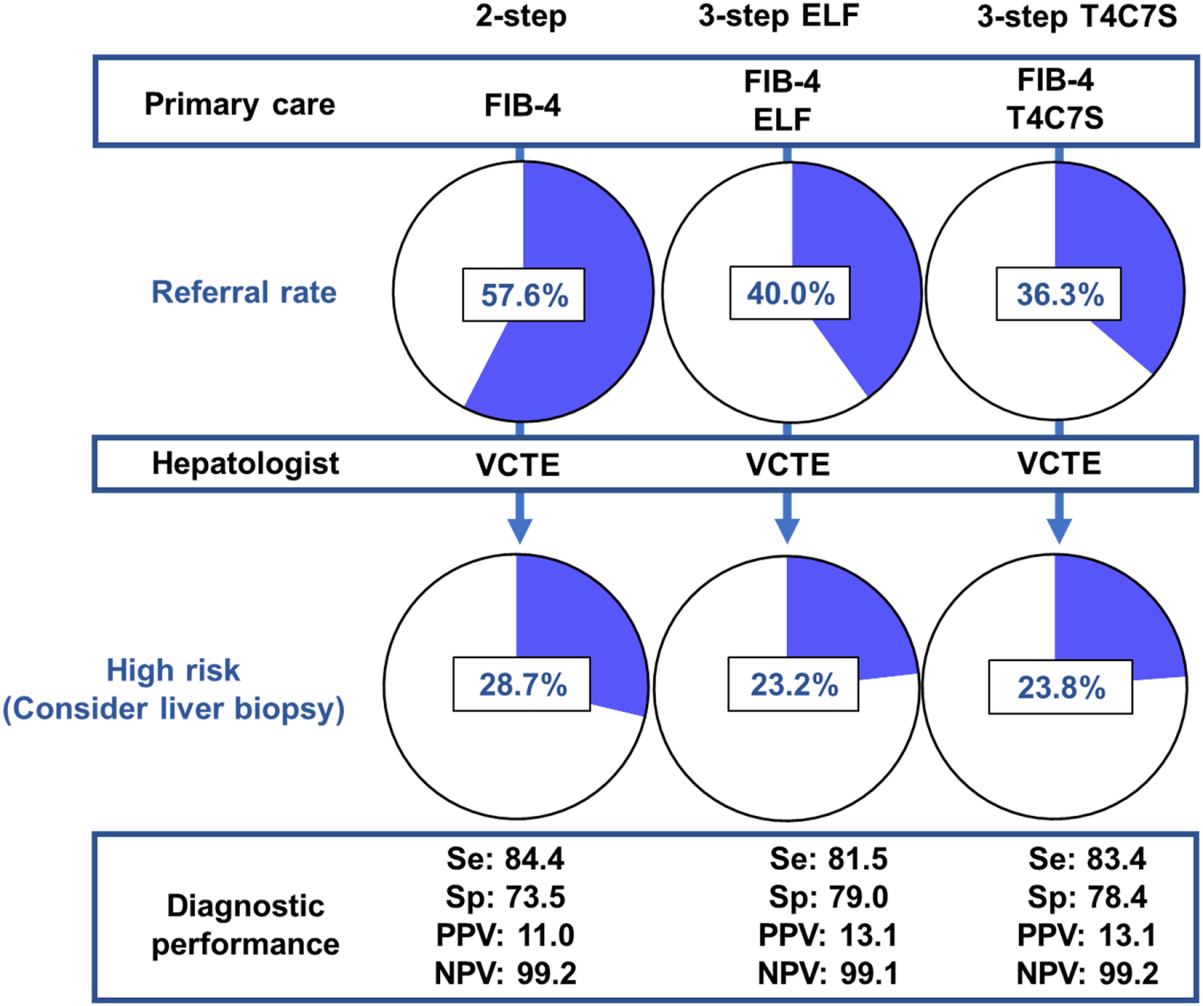
Comparison of each approach applied to a cohort with a low prevalence of advanced fibrosis (3.7%); the 3-step approach can reduce patient referral and liver biopsy rates without losing diagnostic performance. The numbers listed are means. ELF, enhanced liver fibrosis test; FIB-4, Fibrosis-4 index; T4C7S, type IV collagen 7S domain; VCTE, vibration-controlled transient elastography; Se, sensitivity; Sp, specificity; PPV, positive predictive value; NPV, negative predictive value.

In the subpopulation with a low prevalence of advanced fibrosis, which is assumed to match that found in primary care settings, the 3-step approach, compared to the conventional 2-step approach, significantly reduced the number of NAFLD patients referred to specialists and increased the number of patients who could be followed up in primary care clinics. In addition, the 3-step approach significantly reduced the number of patients diagnosed as high risk, that is, those requiring liver biopsy. Furthermore, this approach had a significantly higher specificity and PPV while maintaining a high NPV (Fig. 2). In terms of medical costs, the two types of 3-step approaches with ELF and T4C7S significantly reduced medical costs by 15.8% and 12.5%, respectively, compared to the traditional 2-step approach.

**Figure 3:**
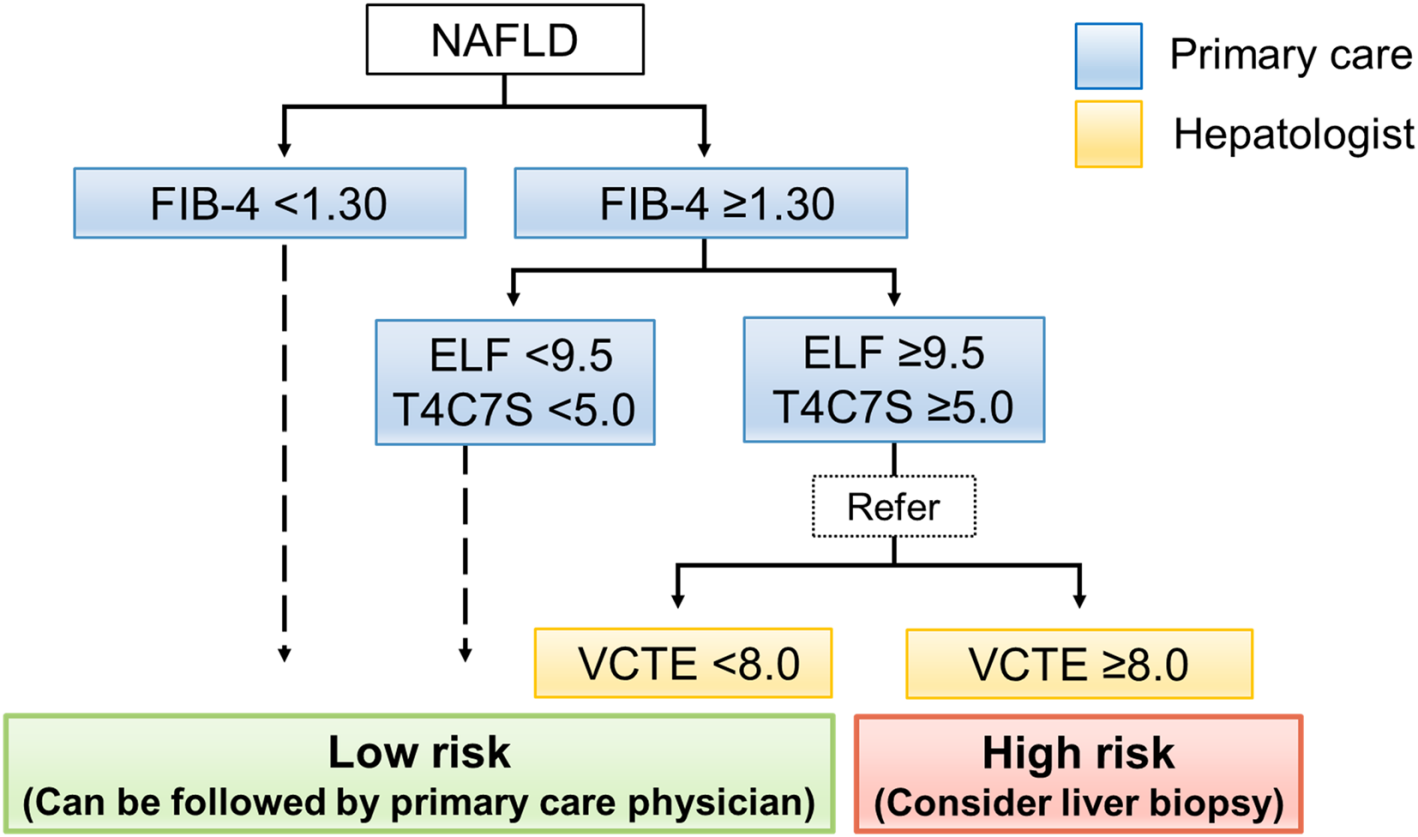
Our novel 3-step approach. The application of this approach will increase the number of patients seen by primary care physicians and decrease the number of specialist referrals. Patients considered at low risk using this approach only need to be followed up by a primary care physician, whereas those considered at high risk need to undergo additional evaluations, including liver biopsy. ELF, enhanced liver fibrosis test; FIB-4, Fibrosis-4 index; T4C7S, type IV collagen 7S domain; VCTE, vibration-controlled transient elastography.

Although FIB-4 is highly useful in assessing NAFLD patients, it has the disadvantage of low PPV. Thus, several 2-step approaches have been developed^5–7,17,20^. Newsome et al. reported that ELF is useful as a second step in a 2-step approach^17^. However, ELF is somewhat expensive and not available in some regions. Thus, we focused on T4C7S, as it has been reported to have a high diagnostic value for predicting advanced fibrosis in NAFLD^12,15,18,21^. It is also of low cost because it can be measured from serum chemiluminescent enzyme immunoassay parameters and does not require a formula because it is a single parameter. Our results showed that T4C7S as a single parameter has a remarkably high predictive capability for advanced fibrosis in NAFLD. Furthermore, T4C7S had comparable diagnostic performance to ELF in the 3-step approach. These results support that T4C7S has the potential to be used as an inexpensive and accurate marker of fibrosis in NAFLD. Our proposed 3-step approach is shown in Fig. 3.

This approach can be practiced by primary care clinics up to the second step. For patients with NAFLD, primary care physicians first assess FIB-4; patients with a FIB-4 higher than 1.3 then undergo ELF or T4C7S. The choice of the second step, ELF or T4C7S, depends on the involved region. If the ELF or T4C7S is above the cutoff value, the patient is referred to a specialist for VCTE. Patients who are considered at low risk based on this approach can be followed up regularly by primary care physicians. In contrast, those considered at high risk will undergo further evaluation by specialists, including liver biopsy. This approach can decrease the rate of unnecessary referrals while improving diagnostic precision, thereby increasing the number of patients that general physicians can follow up on. In addition, this approach may reduce the number of liver biopsies, which are invasive procedures, and may reduce health care costs. For primary care physicians, the 3-step approach may increase their workload due to increased patient visits. However, the 3-step approach would benefit patients by reducing the travel and cost burden and sparing them invasive testing. Additionally, reducing health care costs will be a social benefit.

It is important to reduce the number of false-negative cases in screening tests. In this study, not a few patients in the tertiary care cohort had false-negative results with FIB-4. However, when the FIB-4 is applied to an actual general population cohort with fewer cases of advanced fibrosis, false negatives are expected to be fewer. It may also be possible to reduce the number of false-negative cases by lowering the cutoff for FIB-4 in younger patients^22^. Furthermore, to minimize the damage caused by false negatives, patients diagnosed as low risk should undergo periodic risk assessments.

Clearly, rather than strictly adhering to this approach, referring the patient to a specialist may be better if there are obvious signs of advanced fibrosis or a significant decrease in platelets. Moreover, an approach that considers certain comorbidities, such as metabolic syndrome, may be preferable. These should be the subject of future studies. Furthermore, if VCTE is readily available in some regions, it may be possible to refer patients to a specialized facility after they have undergone VCTE.

This study had some limitations. First, it was based on data from patients who underwent liver biopsy at a tertiary care institution. Therefore, although the bias was corrected using the bootstrap method, the subpopulation with fewer patients with advanced fibrosis may have different characteristics from actual primary care patients. Second, only two centers participated in this study, and all patients were Japanese. Patients in this study appear to have a lower BMI than those in the Western population, which may have affected the accuracy of VCTE. Thus, the generalizability of our 3-step approach in other ethnicities needs to be assessed in multicenter studies. Third, histopathological evaluation of liver biopsy specimens was performed separately by pathologists from each participating institution. Considering the possibility of inter-observer error, diagnosis by an expert pathologist at a central location would have been preferable. However, a standardized scoring system was used. Further large-scale prospective studies are needed to validate our approach.

In this study, we did not establish a validation cohort because we used existing cutoffs to improve diagnostic performance rather than using a new test method or new cutoffs. Instead, we used a bootstrapping approach with repeated resampling in the primary care simulation. In the future, it may be useful to set up an evaluation cohort and a validation cohort for larger studies that can explore cutoff values.

Compared with the conventional 2-step approach, our novel 3-step approach may achieve higher diagnostic performance for advanced fibrosis in NAFLD and lower the rate of unnecessary liver biopsies and specialist referrals. This means that the burden of transportation and invasive procedures on patients can be reduced. Furthermore, this approach may reduce health care costs.

## Methods

### Study subjects

A total of 284 patients who underwent liver biopsy between January 2014 and July 2021 at Yokohama City University Hospital and Saga University Hospital were enrolled in the study. All patients were diagnosed with NAFLD and underwent ELF, T4C7S, and VCTE tests within 6 months before or after liver biopsy. Patients with drinking habits (ethanol consumption > 30 g/day in men and > 20 g/day in women), viral hepatitis (hepatitis B or hepatitis C), drug-induced hepatitis, autoimmune hepatitis, primary biliary cholangitis, sclerosing cholangitis, hemochromatosis, α1-antitrypsin deficiency, or Wilson’s disease were excluded.

### Ethics declarations

This study was approved by Yokohama City University Institutional Review Board and Saga University Clinical Research Review Board and was conducted according to the ethical guidelines of the Declaration of Helsinki. Written informed consent for participation in the study was obtained from each patient.

### Clinical parameters

Demographic, anthropometric, clinical, and laboratory data were collected using standard protocols. Insulin resistance was assessed using the homeostasis model assessment of insulin resistance [immunoreactive insulin (IU/mL) × fasting blood sugar (mg/dL)/405]^23^. The ELF score was determined by analyzing serum samples (Siemens Health Care Diagnostic, Tokyo, Japan) and calculated as follows: 2.278 + 0.851 log (hyaluronic acid) + 0.751 log (N-terminal peptide of procollagen III) + 0.394 log (tissue inhibitor of metalloproteinase-1)^8^. The FIB-4 index was calculated as FIB-4 = [age (years) × AST (IU/L)]/[platelets (10^9^/L) × √ALT (IU/L)]^24^; NAFLD fibrosis score = [1.675 + 0.037] × [age (years) + 0.094] × [BMI (kg/m^2^) + 1.13] × [impaired fasting glucose/diabetes (yes = 1, no = 0) + 0.99] × AST/ALT ratio × 0.013 × platelet count (× 10^9^/L) × 0.66 × albumin (g/dL)^25^; AST/ALT ratio = AST (IU/L)/ALT(IU/L); and AST/platelet ratio = [(AST/upper limit of normal range of AST) × 100]/platelet (10^9^/L).

### VCTE

VCTE (Fibroscan: EchoSens, Paris, France) was performed by experienced operators for liver stiffness measurement. An M-probe was used for patients with a skin-liver capsule distance of less than 25 mm; otherwise, an XL probe was used^6^. Hepatic steatosis was concurrently evaluated with the controlled attenuation parameter using the same device. Data of liver stiffness measurements and controlled attenuation parameters were obtained within 6 months of the liver biopsy. Details of the measurement method have been described previously^26^. The median of ten valid measurements was defined as liver stiffness measurement and controlled attenuation parameters. Cases with interquartile range/median (M) ≥ 30% were excluded.

### Histological assessment

Ultrasound-guided biopsies were performed with 16-gauge needles. Pathology specimens were stained with hematoxylin–eosin and Masson-trichrome or Azan. Histological features were assessed by expert liver pathologists. The fibrosis stage was determined according to the methods proposed by Kleiner et al.^27^ and Brunt et al.^28^. Hepatic steatosis, hepatocyte ballooning, and lobular inflammation were evaluated using the NASH Clinical Research Network scoring system^27^. Advanced fibrosis was defined as fibrosis stage ≥ 3.

### Statistical analysis

A binomial test was used to compare the differences between the 2-step and 3-step approaches for sensitivity, specificity, and proportion diagnosed as high risk. The exact test was used to compare differences between the 2-step and 3-step approaches for PPV and NPV^29^. To simulate the use of each algorithm in a primary care setting with few cases of advanced fibrosis, we constructed a new subpopulation with as few as 3.7% of patients with advanced fibrosis. To maximize the number of patients, all patients without advanced fibrosis were included, and randomly selected patients with advanced fibrosis were added to achieve 3.7%. This is equivalent to the prevalence of advanced fibrosis in the general population of East Asia^5^. In addition, the performance of each approach was compared using the bootstrap method for sensitivity, specificity, PPV, NPV, percentage of patients diagnosed as high risk, and referral rate. All statistical analyses were performed using R version 3.6.3 (R Foundation for Statistical Computing, Vienna, Austria) and JMP 9.0.2 (SAS Institute Inc., Cary, NC, USA).

### Approaches for predicting advanced fibrosis

In the 2-step approach, first, FIB-4 was assessed in all patients, and VCTE was performed as the second step for patients with values higher than the low cutoff, that is, intermediate-to-high FIB-4. The 3-step approach was performed in two ways. First, FIB-4 was performed on all patients. Second, ELF or T4C7S was measured for cases with values higher than the low cutoff for FIB-4. Finally, VCTE was performed for cases with values above the cutoff in the second step. Patients with results below the cutoff in each test were considered at low risk, and patients with results above the cutoff in the final step, VCTE, were considered at high risk. The diagnostic performances of these approaches for advanced fibrosis were evaluated by referring to the liver biopsy results.

### Medical cost estimation

Medical costs were calculated assuming that the 2-step and 3-step approaches were applied in primary care. The mean medical cost per session for each approach was determined using the bootstrap method. ELF is not covered by the Ministry of Health, Labour and Welfare’s medical fee scale in Japan; therefore, the 3-step approach with ELF was compared with the 2-step approach based on UK medical costs. In addition, T4C7S is applicable to the Japanese medical fee points; therefore, the 3-step approach with T4C7S was compared with the 2-step approach based on the Japanese medical costs. Supplementary Tables S2 show the costs for each item used in the calculations, and Supplementary Table S3 shows the cost per round for each approach.

## Supplementary Information

Supplementary Figure S1.

Supplementary Information 2.

## Data Availability

The data that support the findings of this study are available from the corresponding author upon reasonable request.
