# Supplementary figures and images for: A 3-step approach to predict advanced fibrosis in nonalcoholic fatty liver disease: impact on diagnosis, patient burden, and medical costs
Source: Sci Rep. 2022 Oct 28;12:18174. doi: 10.1038/s41598-022-22767-z (PMC9616882; doi:10.1038/s41598-022-22767-z)

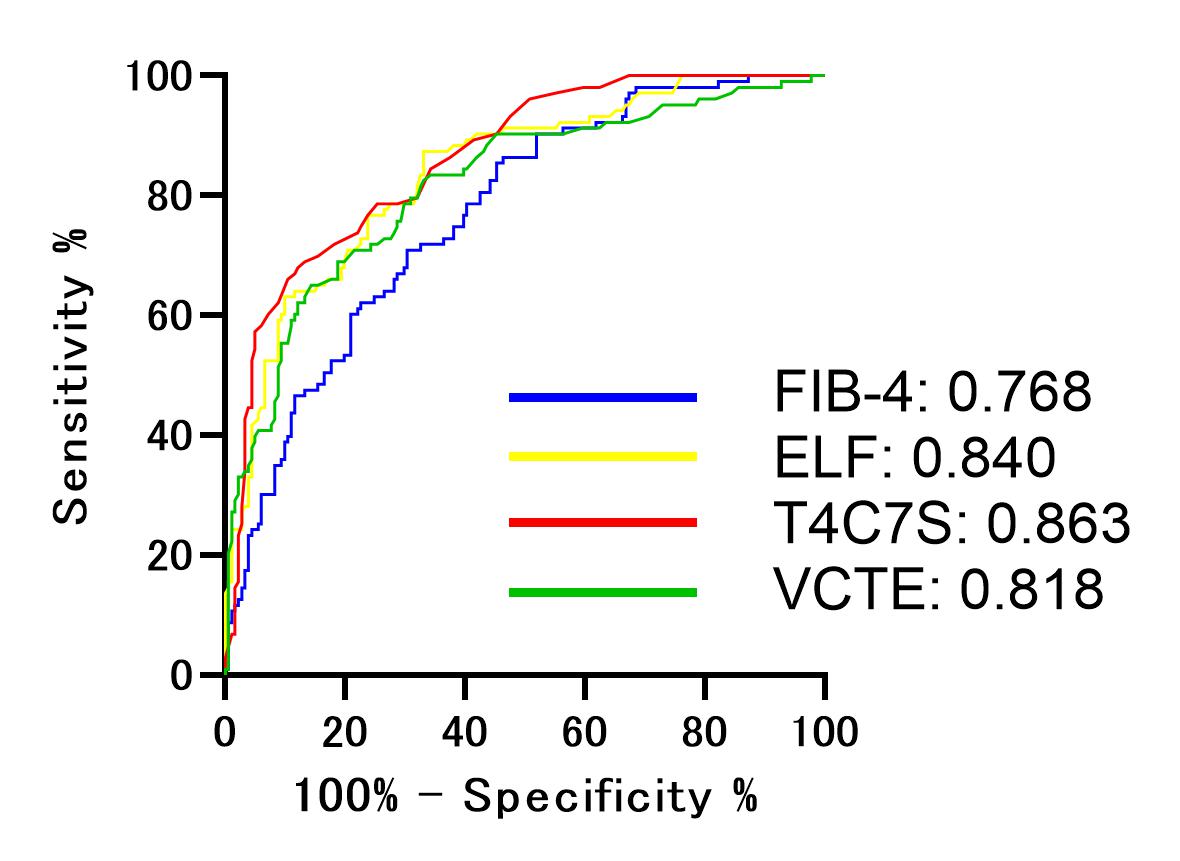

Supplement: Supplementary file 1 — Supplementary Figure S1. [file 41598_2022_22767_MOESM1_ESM.jpg]
