# Supplementary material for: A 3-step approach to predict advanced fibrosis in nonalcoholic fatty liver disease: impact on diagnosis, patient burden, and medical costs
Source: Sci Rep. 2022 Oct 28;12:18174. doi: 10.1038/s41598-022-22767-z (PMC9616882; doi:10.1038/s41598-022-22767-z)
Supplement: Supplementary file 2 — Supplementary Information 2. [file 41598_2022_22767_MOESM2_ESM.docx]

**Supplementary Table S1. Cutoff and diagnostic performance of each test for predicting advanced fibrosis in tertiary care cohort**

|  | AUROC | Cutoff | Se, % | Sp, % | PPV, % | NPV, % |
| --- | --- | --- | --- | --- | --- | --- |
| FIB-4 index | 0.768 | 1.30^*^ | 91.3 | 43.7 | 48.0 | 89.8 |
|  |  | 2.15^†^ | 70.9 | 69.6 | 57.0 | 80.8 |
| ELF score | 0.840 | 9.50^*^ | 93.2 | 43.7 | 48.5 | 91.9 |
|  |  | 9.96^†^ | 87.4 | 66.9 | 60.0 | 90.3 |
|  |  | 7.7^‡^ | 100.0 | 0.6 | 36.4 | 100.0 |
|  |  | 9.8^§^ | 90.3 | 58.0 | 55.0 | 91.3 |
|  |  | 10.51^\|\|^ | 66.0 | 80.7 | 66.0 | 80.7 |
| T4C7S, ng/mL | 0.863 | 5.0^*^ | 97.1 | 49.7 | 52.4 | 96.8 |
|  |  | 6.5^†^ | 68.0 | 88.4 | 76.9 | 82.9 |
| VCTE, kPa | 0.818 | 8.0^*^ | 90.3 | 56.9 | 54.4 | 91.2 |
|  |  | 12.0^†^ | 65.5 | 85.6 | 72 | 81.2 |
| Plt, ×10^3^/μL | 0.707 | 176^†^ | 61.2 | 75.7 | 58.9 | 77.4 |
| AAR | 0.723 | 0.925^†^ | 64.1 | 72.9 | 57.4 | 78.1 |
| APRI | 0.725 | 0.838^†^ | 76.7 | 60.8 | 52.7 | 82.1 |
| NFS | 0.740 | -0.826^†^ | 76.7 | 59.7 | 52.0 | 81.8 |

*Cutoff used in algorithms; †maximal Youden index; ‡low and §high thresholds recommended by Siemens; ||high threshold recommended by NICE guideline.

AAR, aspartate aminotransferase-to-alanine transaminase ratio; APRI, aspartate aminotransferase to platelet ratio index; FIB-4, Fibrosis-4 index; NFS, non-alcoholic fatty liver disease fibrosis score; VCTE, vibration-controlled transient elastography; ELF, enhanced liver fibrosis test; T4C7S, type IV collagen 7S domain; Se, sensitivity; Sp, specificity; PPV, positive predictive value; NPV, negative predictive value.

**Supplementary Table S2. Items used in the calculation of medical costs**

**a) Medical cost in the UK**

|  | Cost, pound | Source |
| --- | --- | --- |
| GP (10 minutes) | 3.9 | PSSRU Unit Costs of Health and Social Care |
| FIB-4 | 4.52 | Personal communication Royal Free laboratories |
| ELF | 42 | North Middlesex |
| Consultant hepatologist | 176 | Department of Health reference costs  First attendance face to face (Code 306) |
| VCTE | 47 | Department of Health reference costs 2013-14 (Code RA23Z) |
| Liver biopsy | 956.61 | Stevenson et al. Health Technol Assess. 2012;16(4):1-174. |

ELF, enhanced liver fibrosis test; FIB-4, Fibrosis-4 index; GP, General practitioner; VCTE, vibration-controlled transient elastography

**b) Medical cost in Japan**

|  | Cost, yen | Source; Codes set by Japan’s Ministry of Health, Labor and Welfare |
| --- | --- | --- |
| First visit fee | 2,880 | A000 |
| FIB-4 | 450 | - |
| AST | 170 | D007 |
| ALT | 170 | D007 |
| PLT | 210 | D005 |
| T4C7S | 1,440 | D007 |
| Patient referral document | 2,500 | B009 |
| VCTE | 2,000 | D215-2 |
| Liver biopsy | 16,000 | D412 |

ALT, alanine transaminase; AST, aspartate aminotransferase; ELF, enhanced liver fibrosis test; FIB-4, Fibrosis-4 index; GP, General practitioner; PLT, platelet count; T4C7S, type IV collagen 7S domain; VCTE, vibration-controlled transient elastography

**Supplementary Table S3. Costs for each approach**

a) 2-step in the UK

|  | Cost, pound | Input parameters |
| --- | --- | --- |
| FIB-4 (low) | 43.52 | GP, FIB-4 |
| FIB-4 (high)–VCTE (low) | 266.52 | GP, FIB-4, Consultant hepatologist, VCTE |
| FIB-4 (high)–VCTE (high) | 1,223.13 | GP, FIB-4, Consultant hepatologist, VCTE, Liver biopsy |

b) 3-step ELF in the UK

|  | Cost, pound | Input parameters |
| --- | --- | --- |
| FIB-4 (low) | 43.52 | GP, FIB-4 |
| FIB-4 (high)–ELF (low) | 85.52 | GP, FIB-4, ELF |
| FIB-4 (high)–ELF (high)–VCTE (low) | 308.52 | GP, FIB-4, ELF, Consultant hepatologist, VCTE |
| FIB-4 (high)–ELF (high)–VCTE (high) | 1,265.13 | GP, FIB-4, ELF, Consultant hepatologist, VCTE, Liver biopsy |

c) 2-step in Japan

|  | Cost, yen | Input parameters |
| --- | --- | --- |
| FIB-4 (low) | 3,330 | First visit fee, FIB-4 |
| FIB-4 (high)–VCTE (low) | 10,710 | First visit fee×2, FIB-4, Patient referral document, VCTE |
| FIB-4 (high)–VCTE (high) | 26,710 | First visit fee×2, FIB-4, Patient referral document, VCTE, Liver biopsy |

d) 3-step-T4C7S in Japan

|  | Cost, yen | Input parameters |
| --- | --- | --- |
| FIB-4 (low) | 3,330 | First visit fee, FIB-4 |
| FIB-4 (high)–T4C7S (low) | 4,770 | First visit fee, FIB-4, T4C7S |
| FIB-4 (high)–T4C7S (high)–VCTE (low) | 12,150 | First visit fee×2, FIB-4, Patient referral document, T4C7S, VCTE |
| FIB-4 (high)–T4C7S (high)–VCTE (high) | 28,150 | First visit fee×2, FIB-4, Patient referral document, T4C7S, VCTE, Liver biopsy |

ELF, enhanced liver fibrosis test; FIB-4, Fibrosis-4 index; GP, General practitioner; T4C7S, type IV collagen 7S domain; VCTE, vibration-controlled transient elastography

**Supplementary Figure S1**

Receiver operating characteristic (ROC) curves for the diagnosis of fibrosis stage ≥3 of FIB-4 (blue line), ELF (yellow line), T4C7S (red line), and VCTE (green line). The area under ROC of ELF, T4C7S and VCTE were >0.800.

FIB-4, Fibrosis-4 index; VCTE, vibration-controlled transient elastography; ELF, enhanced liver fibrosis test; T4C7S, type IV collagen 7S domain
